# New Developments in the Pathophysiology and Management of Diabetic Retinopathy

**DOI:** 10.1155/2013/424258

**Published:** 2013-12-10

**Authors:** Ahmed M. Abu El-Asrar, Edoardo Midena, Mohamed Al-Shabrawey, Ghulam Mohammad

**Affiliations:** ^1^Department of Ophthalmology, College of Medicine, King Saud University, P.O. Box 245, Riyadh 11411, Saudi Arabia; ^2^Department of Ophthalmology, Padova University Hospital, Via Giustiniani 2, 35128 Padova, Italy; ^3^Medical College of Georgia and College of Graduate Studies at the Georgia Health Sciences University, Augusta, GA 30912, USA

Diabetes mellitus is a chronic disorder of glucose metabolism with serious multisystem complications including microvascular dysfunction such as retinopathy, nephropathy, and neuropathy. The prevalence of diabetes has been rising in the last few decades and affecting working age population which reflects the socioeconomic burden on different communities and a major challenge to the current health care. Diabetic retinopathy (DR), which is a slow-progressing and multifactorial complication of diabetes, is the most common cause of vision loss in patients with diabetes. According to the World Health Organization, in the world, about more than 371 million people have diabetes and approximately 5 million individuals have diabetic retinopathy, accounting for 5 percent of world blindness. In spite of all advances in the understanding of chronic diabetic complications, DR remains a significant clinical problem partially due to lack of effective therapeutic intervention. The current therapy is mostly invasive intervention and mainly prevents the progression of the disease but does not effectively restore the lost vision. Therefore, there is an urgent need to understand in detail the underlying molecular and cellular mechanisms of the DR and also to advance the diagnostic tools to diagnose the early microvascular and neuronal changes during DR.

The current special issue through a number of experts in the field of DR presents both review and original research articles that provide an overview of the work conducted to date and expand our understanding of the underlying mechanism of DR. The articles include discussion of the mechanism of retinal neovascularization (angiogenesis, vasculogenesis) and neurodegeneration including the role of enhanced inflammatory pathways, cellular signaling, and oxidative stress. Each of the papers in this series is briefly highlighted below.

The improvement of diagnostic technology has revolutionized the clinical approach to eye disorders, mainly retinal diseases. Optical coherence tomography (OCT) has introduced clinicians to a sort of *in vivo* retinal histology, particularly with the use of spectral domain OCT. With spectral domain OCT, we are not yet at cellular level, but we may obtain and discuss individual retinal layers. The clinical use of spectral domain OCT has largely contributed to the understanding of pathophysiology of diabetic retinopathy, both in preclinical and clinical phases. Two papers of this special issue dedicated to diabetic retinopathy clearly show how relevant is the contribution of spectral domain OCT in the quantification of retinal layer changes secondary to diabetes. T. Murakami and N. Yoshimura offer a well-illustrated and updated review of the inner and outer retinal changes in diabetic macular edema, the most important cause of legal blindness among diabetics. They discuss the relevance of any OCT detail to improve our knowledge about the pathophysiology of diabetic macular edema and stress the importance of considering these details as predictive factors of treatment results. They also point to the importance of the still poorly known role of the choroid, which may be measured by OCT, in the pathophysiology of diabetic retinopathy. S. Vujosevic and E. Midena not only confirm the importance of the study of retinal layers, quantified by OCT, in nonproliferative diabetic retinopathy, but also illustrate the precocious changes of retinal layers in preclinical retinopathy. This means that OCT may contribute to detection of subtle retinal changes before an overt microvasculopathy develops. These changes are visible mainly in the inner retina, with a speculated involvement of the Müller cells, never reported before. In addition, S. Vujosevic and S. Midena demonstrated using spectral domain OCT the presence of retinal hyperreflective spots (HRS) in diabetic eyes even when clinical retinopathy is undetectable. Their number increases with progressing retinopathy. HRS are initially mainly located in the inner retina, where resident microglia is present. With progressing retinopathy, HRS reach the outer retinal layer. HRS may represent a surrogate of microglial activation in diabetic retina.

The paper by A. M. Abu El-Asrar et al. describes levels of angiogenic and endothelial progenitor cell mobilizing (vasculogenic) factors in vitreous fluid from proliferative diabetic retinopathy (PDR) patients and correlates their levels with clinical disease activity. The pathophysiology of DR involves multiple molecular pathways and is characterized by retinal neovascularization. Retinal neovascularization (RNV) in PDR occurs mainly through angiogenesis. However, increasing evidence suggests that vasculogenesis, the de novo formation of blood vessels from circulating bone-marrow-derived endothelial progenitor cells (EPCs), also contributes to retinal neovascularization. Recent studies have shown that circulating bone-marrow-derived EPCs home to the ischemic region, differentiate into mature endothelial cells in situ, and can contribute to the process of neovascularization. Angiogenesis and vasculogenesis are dependent on several cytokines/chemokines and their associated tyrosine kinase receptors. Abu El-Asrar et al.'s findings suggest that the upregulation of VEGF, sVEGFR-2, SCF and s-kit in the vitreous fluid from patients with PDR reflects angiogenesis and vasculogenesis in PDR.

Growing body of evidence supports the hypothesis that damaging effect of elevated glucose in the retina may, in part, be due to its ability to increase mitogen-activated protein kinases (MAPK) signaling pathway in the retina. Extracellular-signal-regulated kinases-1/2 (ERK1/2) are the most extensively characterized members of MAPK family proteins. G. Mohammad et al. describe the role of ERK1/2 signaling pathway in the activation of inflammatory mediators in the diabetic retina. The authors' data provide evidence that ERK1/2 signaling pathway is involved in MMP-9, iNOS, IL-6, and TNF-*α* induction in diabetic retinas and suggest that ERK1/2 can be a novel therapeutic target in diabetic retinopathy.

Diabetes is known to cause oxidative stress in the retina, and this abnormality results from increased generation of reactive oxygen species (via mitochondria and NADPH oxidase) and decreased activity of antioxidant enzymes. Considering that oxidative stress represents the key factors in the onset and progression of diabetic retinopathy, antioxidant and anti-inflammatory products are expected to produce significant therapeutic advantages.

The paper by G. Bucolo et al. describes that treatment of diabetic rats with the fortified extract of red berries, *Ginkgo biloba*, and white willow bark (containing carnosine and *α*-lipoic acid) significantly lowered retinal cytokine levels (TNF-*α*,VEGF) and suppressed diabetes-induced lipid peroxidation.

Diabetes-induced oxidative stress disturbs retinal homeostasis by activating glial cells, reducing neurotrophic support, and increasing proinflammatory cytokines. Recent studies proved that neurotrophins including nerve growth factor (NGF) are emerging as critical mediators of DR. NGF activates two different receptors including the high affinity tropomyosin-related receptor A (TrkA), which is a tyrosine kinase, and the low affinity p75NTR. The paper by S. L. Elshaer et al. demonstrated that the angiogenic response of NGF was mediated via activation of TrkA suggesting that the proNGF can contribute to PDR at least in part via activation of TrkA.

We sincerely hope that the present special issue may provide useful information to understand the mechanisms, the clinical effects, and the novel treatments in the development of DR.

*Ahmed  M.  Abu  El-Asrar* *Ahmed  M.  Abu  El-Asrar* *Edoardo  Midena* *Edoardo  Midena* *Mohamed  Al-Shabrawey* *Mohamed  Al-Shabrawey* *Ghulam  Mohammad* *Ghulam  Mohammad*

